# ^18^Fluorine-fluorodeoxyglucose PET/CT Imaging in Childhood Malignancies

**DOI:** 10.4274/mirt.galenos.2020.64436

**Published:** 2021-02-09

**Authors:** Nilüfer Bıçakçı, Murat Elli

**Affiliations:** 1 University of Health Sciences Turkey, Samsun Training and Research Hospital, Clinic of Nuclear Medicine, Samsun, Turkey; 2 İstanbul Medipol University Faculty of Medicine, Department of Pediatric Oncology, İstanbul, Turkey

**Keywords:** 18F-FDG PET/CT, childhood malignancy, staging, restaging, response

## Abstract

**Objectives:**

The aim of the study was to evaluate the utility of ^18^fluorine-fluorodeoxyglucose (^18^F-FDG) positron emission tomography/computed tomography (PET/CT) in the diagnosis, staging, restaging, and treatment response of childhood malignancies.

**Methods:**

This study included 52 patients (32 boys, 20 girls) who were referred to our clinic between November 2008 and December 2018 with the diagnosis of malignancy. The patients were evaluated retrospectively. Median age of the patients was 13 years (range 2-17). ^18^F-FDG was given to the patients intravenously, and time of flight with PET/16 slice CT was performed 1 hour thereafter. The lowest dose was 2 mCi (74 MBq) and the highest dose was 10 mCi (370 MBq). Fasting blood sugars of all patients were found below 200 mg/dL (11.1 mmol/L).

**Results:**

^18^F-FDG PET/CT was performed to evaluate the response to treatment in 38 of 52 children, staging in 11 patients (staging and evaluation of the response to treatment in nine of them), restaging in 2 patients, restaging, and evaluation of the response to treatment in 1 patient. ^18^F-FDG PET/CT examination was reported as normal in 13 patients (5 girls, 8 boys). The pathological ^18^F-FDG uptake was detected in 39 patients (14 girls, 25 boys), which indicated metastasis and/or recurrence of the primary disease. Total number of deaths was 30 (13 girls, 17 boys).

**Conclusion:**

^18^F-FDG PET/CT has a significant role for staging, restaging, treatment response, and detection of metastatic disease but it is limited for the early diagnosis of childhood cancers

## Abstract (tr)

**Amaç:**

Çalışmamızın amacı, çocukluk çağı malignitelerinin tanı, evreleme, yeniden evreleme ve tedaviye cevabın değerlendirilmesinde ^18^flor-florodeoksiglukoz (^18^F-FDG) pozitron emisyon tomografisi/bilgisayarlı tomografinin (PET/BT) yararını göstermektir.

**Yöntem:**

Kasım 2008 ve Aralık 2018 tarihleri arasında, malignensi tanılı 52 hastanın (32 erkek, 20 kız) dosyaları ve görüntüleri geriye dönük olarak incelendi. Ortalama yaş 13 (2-17) idi. ^18^F-FDG’nin intravenöz enjeksiyonundan 1 saat sonra, time of flight/16 kesit BT yapıldı. Çalışmamızda en düşük doz 2 mCi (74 MBq), en yüksek doz 10 mCi (370 MBq). Tüm hastaların açlık kan şekerleri 200 mg/dL’nin (11,1 mmol/L) altındaydı.

**Bulgular:**

^18^F-FDG PET/BT, 52 hastanın 38’ine tedaviye yanıt değerlendirilmesi, 11 hastaya evreleme (9 hasta evreleme ve aynı zamanda tedaviye yanıt değerlendirilmesi), 2 hastaya yeniden evreleme, 1 hastaya yeniden evreleme ve tedaviye yanıt değerlendirilmesi amacıyla yapıldı. ^18^F-FDG PET/BT çalışması 13 hastada (5 kız, 8 erkek) normaldi. Otuz dokuz hastada (14 kız, 25 erkek) çalışma, metastaz ve/veya primer hastalığın nüksü ile uyumlu bulundu. Toplam ölüm sayısı 30 (13 kız, 17 erkek) idi.

**Sonuç:**

^18^F-FDG PET/BT çocukluk çağı malignensilerinin tanı, evreleme, yeniden evreleme ve tedaviye yanıt değerlendirilmesi açısından çok faydalıdır ancak erken tanıda yararı sınırlıdır.

## Introduction

^18^Fluorine-fluorodeoxyglucose (^18^F-FDG) positron emission tomography/computed tomography (PET/CT) plays an important role for diagnosis, staging, restaging, response to treatment, and evaluation of prognosis in childhood malignancies (1,2). PET-only examinations have been replaced by hybrid systems in the recent decades, where PET and CT are used together in oncology (3). In this imaging system, PET and CT are used together for functional data and morphological information, respectively (4). ^18^F-FDG PET/CT is also known to have high sensitivity and specificity (86% and 80%, respectively) in childhood malignancies (5,6,7).

The type of childhood malignancies varies according to the age groups. The most common childhood malignancy is leukemia with a rate of 30%; other malignancies are brain tumors (20%), lymphomas (14%), neuroblastoma (7%), soft tissue sarcomas (7%), Wilms’ tumor (6%), bone tumors (5%), germ cell tumors (3%), melanoma (3%), hepatic tumors (1%), etc. Lymphoma and germ cell tumors are more common in children between the ages of 14 and 19 years (8,9,10,11,12,13,14). The childhood tumors in which ^18^F-FDG PET/CT is used frequently include lymphomas, brain tumors, soft tissue sarcomas, neuroblastoma, Wilms’ tumor, germ cell tumors, and neurofibromatosis 1 (15). The most commonly used radionuclides in nuclear medicine for the cancer imaging are gallium-67 (^67^Ga) citrate, thallium-201 chloride, technetium-99m sestamibi, and ^18^F-FDG. ^18^F-FDG causes lower radiation exposure due to relatively short half-life (110 minutes), and it is also a widely available radionuclide agent (2). ^18^F-FDG mimics glucose in cell uptake process and thus acts as a marker of glucose usage. ^18^F-FDG is not a tumor-specific agent and can be kept in cells in case of many physiological and pathological conditions. Dual-time-point imaging can help to increase the specificity of ^18^F-FDG imaging (3).

We evaluated the role of ^18^F-FDG PET/CT in diagnosis, staging, restaging, treatment response, and detection of metastatic disease of childhood malignancies in this study.

## Materials and Methods

Fifty-two children (32 boys, 20 girls) with tissue-confirmed malignancies underwent ^18^F-FDG PET/CT examination between November 2008 and December 2018. The median age of the patients was 13 years (range 2-17 years). The study was approved by the University of Health Sciences Turkey, Samsun Training and Research Hospital of Local Ethics Committee (protocol number: GOKA/2020/10/6).

All imaging studies were performed under at least 4 hours of total fasting. The dose of ^18^F-FDG was calculated as 0.15 mCi/kg (5.55 MBq/kg) between 2008 and 2010. After 2010, it was calculated according to the radiopharmaceutical doses published in the 2016 North American Consensus Guidelines, which has been updated as the whole-body ^18^F-FDG with 3.7-5.2 MBq/kg (0.1-0.4 mCi/kg), and the minimum dose was recommended as 37 MBq (1 mCi). In our study, the lowest dose was 2 mCi (74 MBq), and the highest dose was 10 mCi (370 MBq). Fasting blood sugar level of all patients was found to be less than 200 mg/dL (11.1 mmol/L). CT parameters were obtained with ultra-low dose (80 kVp, 5 mAs, and 1.5:1 pitch). After 45-60 minutes from application of ^18^F-FDG, CT images were obtained for attenuation correction without intravenous contrast, and then PET images were gathered. ^18^F-FDG examination was performed with time of flight PET/16 section CT (Philips Gemini TF), and the PET detector crystal material was LYSO.

Sedation was used in 6 patients who were under 8 years of age during the ^18^F-FDG PET/CT examination. We used the oral chloral hydrate as 50-70 mg/kg for young children less than 15 kg of body weight, according to application guide of the American Academy of Pediatrics (16,17). This dosage is appropriate in most nuclear medicine applications. In our study it was sufficient for the younger age group.

Brown adipose tissue produces heat in case of exposure to cold and causes focal increased ^18^F-FDG uptake and may mimic muscle or malignancy (18,19,20). However, diazepam was not used in any of our patients as the waiting room temperatures were ensured to be high enough to prevent cold exposure in our clinic.

^18^F-FDG PET/CT indications and findings of the patients were analyzed retrospectively. Patient characteristics are listed in Table 1.

No statistical analysis was performed.

## Results

^18^F-FDG PET/CT was applied to 52 children for evaluation of response to treatment in 38, staging in 11 (2 staging and nine staging and evaluating response to treatment), restaging in 2, evaluation of response to treatment with restaging in 1 patient.

**Figure 1:**
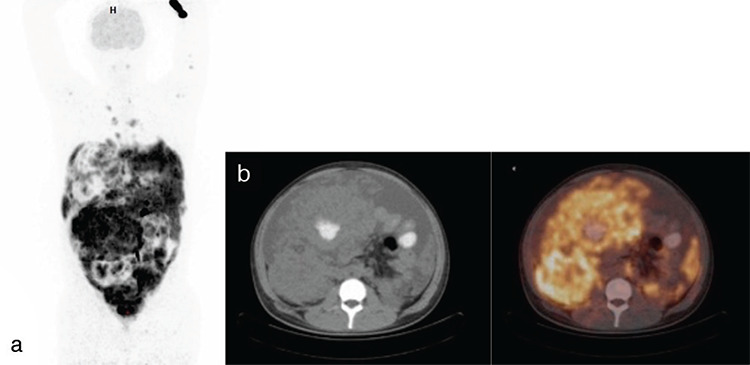
MIP (a), transaxial CT (b), and fusion ^18^F-FDG PET/CT images of a 15-year-old male patient. Abdominal lymph node biopsy revealed a high-grade malign B-cell lymphoma (Burkitt’s lymphoma). Multiple hypermetabolic mediastinal, abdominal, pelvic lymph nodes, massive abdominal fluid, and bone marrow involvement were seen on ^18^F-FDG PET/CT imaging ^18^F-FDG: ^18^Fluorine-fluorodeoxyglucose, PET: Positron emission tomography, CT: Computed tomography, MIP: Maximum intensity projection

Twenty-three patients had the diagnosis of lymphoma [14 non-Hodgkin’s lymphoma (NHL), 9 HL], and ^18^F-FDG PET/CT was performed for staging and response to treatment in 10, for response to treatment in 11, and for restaging in 2 patients. ^18^F-FDG PET/CT detected more nodal lesions than CT in 10 staged patients. Detection of multiple lesions in the skeletal system and bone marrow increased the stage in these patients (Figure 1).

Patients with Ewing’s sarcoma (ES), rhabdomyosarcoma, neuroblastoma, malignant melanoma, malignant mesenchymal tumor, retinoblastoma, nasopharynx carcinoma, and germ cell tumors did not undergo ^18^F-FDG PET/CT study before treatment, and ^18^F-FDG PET/CT was performed after treatment to evaluate the response to treatment. Metastatic disease was detected by ^18^F-FDG PET/CT in the bone, liver, brain, and abdominal and mediastinal lymph nodes of the patients with neuroblastoma (n=7) during follow-up.

Seven patients with ES and one with peripheric primitive neuroendocrine tumor were evaluated with ^18^F-FDG PET/CT for local and systemic involvement after chemotherapy. Three local recurrences and five abdominal/inguinal metastatic lymph nodes were detected with the ^18^F-FDG PET/CT. In patients with rhabdomyosarcoma, ^18^F-FDG PET/CT detected three recurrent diseases and one metastatic disease on follow-up after adjuvant therapy (one had Li-Fraumeni syndrome).

**Figure 2:**
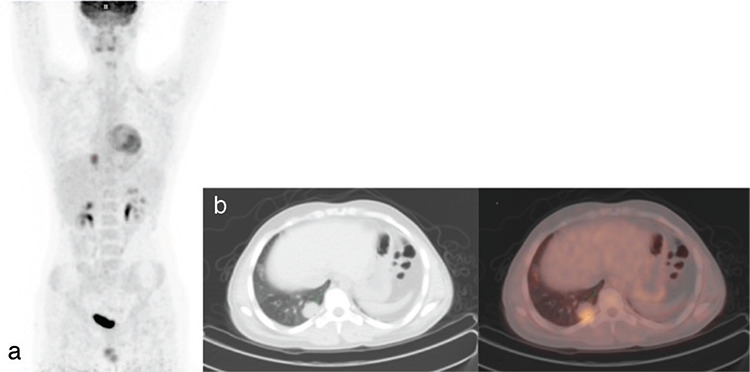
MIP (a), transaxial CT (b), and fusion ^18^F-FDG PET/CT images of a 16-year-old male patient. Histopathologically, diagnosis was rhabdomyosarcoma. Hypermetabolic metastatic nodule was seen in the right lung posterobasal segment on ^18^F-FDG PET/CT imaging ^18^F-FDG: ^18^Fluorine-fluorodeoxyglucose, PET: Positron emission tomography, CT: Computed tomography, MIP: Maximum intensity projection

^18^F-FDG PET/CT was performed for evaluation of treatment response in 2 patients with testicular carcinoma. In the other patient, ^18^F-FDG PET/CT was performed for restaging, and a lung metastasis was detected (Figure 2).

No recurrence or metastasis was identified in ^18^F-FDG PET/CT of 13 patients. Thirty patients died on follow-up; 7 patients had NHL, and the other 23 patients had ES (n=8), neuroblastoma (n=7), rhabdomyosarcoma (n=1), malignant mesenchymal tumor (n=1), germ cell tumor (n=1), immature teratoma (n=1), and retinoblastoma (n=1) (Table 2).

## Discussion

Our findings indicate that ^18^F-FDG PET/CT is an essential imaging modality and provided important information for diagnosis, staging, restaging, evaluation of the response to treatment, and detection of metastatic disease. However, this study is limited in early diagnosis of childhood malignancies.

Although childhood malignancies are relatively rare as compared to adults, still they are a significant cause of mortality and constitute the second most frequent cause of death after trauma in children (21). Leukemia accounts for more than half of all childhood solid tumors, and the other frequent childhood cancers are brain tumors, lymphomas, neuroblastoma, soft tissue sarcomas, Wilms’ tumor, and bone tumors (8,21).

Childhood cancers differ from adults in terms of epidemiology, histological patterns, clinical behavior, treatment response, and prognosis. Appropriate treatment reduces the mortality rate. Early and correct diagnosis is essential. Improved oncological results lead to an increased incidence of late complications of childhood cancers. ^18^F-FDG PET/CT as an imaging technique is well studied in adults. ^18^F-FDG PET/CT is increasingly used for staging, prognosis, determination of biopsy location, evaluation of treatment response, radiotherapy planning, and follow-up in many types of childhood cancers (5,22,23,24,25,26,27,28). The role of ^18^F-FDG PET/CT is, however, limited for the early diagnosis of childhood cancers but has a significant role for staging, treatment response, and detection of metastatic disease. Thus, ^18^F-FDG PET/CT has been used increasingly in children with malignancy for these features.

^18^F-FDG is the most commonly used radiopharmaceutical in PET for oncological purposes. ^18^F-FDG is a cyclotron radiopharmaceutical with a half-life of 110 minutes. ^18^F-FDG is a glucose analog and is transported into the cell by glucose transporters and often participates in the first stage of the physiological glycolytic pathway. Therefore, the degree of ^18^F-FDG uptake indicates the metabolic activity of the cells (29). Evaluation after treatment with therapeutic agents does not affect tumor size immediately but inhibits tumor metabolism and proliferation. So, accumulation of ^18^F-FDG in metabolically active tumor cells has revolutionized oncological imaging. Although this discovery was made several decades ago, the ability of ^18^F-FDG PET imaging for differentiation of active/stable disease and to provide more clinical information than the simple anatomical localization of the disease has been appreciated recently.

New generation PET devices are faster and have higher resolution. ^18^F-FDG PET reflects both the metabolic status and the proliferative potential of the disease in patients receiving either conventional or experimental therapy. ^18^F-FDG PET can be used in the majority of childhood cancers as convenient as CT and magnetic resonance imaging (MRI) (30,31,32,33). Metabolic changes induced by chemotherapy occur before morphological changes. Since the ^18^F-FDG intake provides direct measurement of tumor glucose metabolism, the tumor’s response to treatment can be evaluated earlier before the tumor shrinks. The response to treatment may also be predicted more accurately than conventional techniques (34,35,36,37). In our study, we also used ^18^F-FDG as imaging radiopharmaceutical in all pediatric patients. We adjusted the radiopharmaceutical doses in children in line with the 2016 North American Consensus Guidelines renewed in 2010 and later (38,39).

Lymphomas are the third most common type of tumor in the childhood group that account for 14% of all cancer cases. While NHL is more commonly found in young children, HL is more common in the adolescent group. ^18^F-FDG PET/CT is used for staging, evaluation of treatment response, and relapse of disease, before bone marrow or stem cell transplantation for diagnostic and prognostic information in children (40). London et al. (41) in their study compared conventional imaging methods (CT, ultrasonography, MRI, and bone scintigraphy) with ^18^F-FDG PET/CT in pediatric patients diagnosed with HL and NHL to differentiate malignant lesion and to predict poor response to treatment. The sensitivity, specificity, and accuracy (95.9%, 99.7%, and 99.6%, respectively) of ^18^F-FDG PET/CT were found to be higher than other conventional imaging methods (70.1%, 99.0%, and 98.3%, respectively) for lymphoma in children. In a study by Cheng et al. (6), ^18^F-FDG PET/CT detected lesions that could not be detected by CT in 50% of children with HL and 42.9% of children with NHL. In our study ^18^F-FDG PET/CT detected more nodal lesions than CT in 10 patients (50% of children with HL and 50% of children with NHL). The stage of malignancy was also increased because of additional lesions in the skeletal system and bone marrow in these patients.

Tumors of sympathetic nervous system constitute about 7% of all childhood tumors, and neuroblastoma is the most common tumor in this group (42). Approximately 10% of neuroblastomas do not uptake metaiodobenzylguanidine (MIBG), and ^18^F-FDG PET/CT can be used in the evaluation of MIBG-negative patients (42,43,44). Another study reported that MIBG scintigraphy and ^18^F-FDG PET/CT were equally effective for patients with distant disease in demonstrating bone metastases after primary tumor resection and chemotherapy (45). Choi et al. (46) showed that ^18^F-FDG PET/CT is more sensitive than CT for evaluation of distant lymph node metastases and can detect recurrent lymph node metastases. Similarly, bone, liver, brain, and widespread lymph node metastases in the abdomen and mediastinum were detected by ^18^F-FDG PET/CT in our patients with neuroblastoma after the adjuvant therapy. Other alternative diagnostic imaging technique in neuroblastoma without MIBG uptake has been investigated including radiolabeled somatostatin analogs such as octreotide and DOTA-conjugated peptides [e.g., ^68^Ga DOTATATE (DOTA0-Try3) octreotate], ^68^Ga DOTATOC (DOTA0-Try3) octreotide, and ^68^Ga DATANOC (DOTA0-1NaI3) octreotide. These analogs can bind selectively to somatostatin receptors 2 (47). DOTA-peptides can also be labeled with beta-emitting isotopes, for example, ^177^Lu or ^90^Y, to provide peptide receptor radionuclide therapy for neuroendocrine tumors in adults (48,49,50,51,52,53,54) and have been used in small studies with relapsed neuroblastoma in children (55,56,57,58).

ES is a heterogenous tumor including ES of the bone, extraosseous ES, and peripheral primitive neuroectodermal tumor. It is the second most common bone malignancy in the pediatric age group, and its incidence among all childhood cancers is approximately 3% (59). Like many other malignant tumors, ES has an increased glycolysis rate, and as a result, it shows increased ^18^F-FDG accumulation. ^18^F-FDG PET/CT is particularly useful in detecting, staging, and restaging of the bone metastases in musculoskeletal tumors and often provides important additional information that may alter the treatment plan (60). Seven patients with ES and one patient with peripheral primitive neuroectodermal tumor were evaluated with ^18^F-FDG PET/CT for local and systemic disease after chemotherapy in our study. Three local recurrences and five abdominal/inguinal metastatic lymph nodes were detected with the ^18^F-FDG PET/CT.

Rhabdomyosarcoma is responsible for 4%-8% of malignant diseases in children under 15 years of age (2). Although most of the cases are sporadic, some related congenital and genetic diseases are reported (61). One of our four rhabdomyosarcoma patients had Li-Fraumeni syndrome. ^18^F-FDG PET/CT detected three recurrent and one metastatic disease on follow-up after treatment of rhabdomyosarcoma. There are few studies in the literature on the role of ^18^F-FDG PET/CT in treatment response evaluation in childhood rhabdomyosarcoma. Eugene et al. (62) reported that ^18^F-FDG PET/CT predicted the treatment response better than conventional imaging methods in a study group of 23 patients after 3 cycles of treatment. They also had demonstrated 69% complete radiological response with ^18^F-FDG PET/CT while it was reported as 8% in conventional methods. This finding supports that the metabolic response of the treatment occurred earlier than the response in tumor size. ^18^F-FDG PET/CT was also performed in our clinic for evaluating response to treatment in patients with malignant mesenchymal tumor, testicular tumors, retinoblastoma, immature teratoma, nasopharyngeal cancers, and germ cell tumors. ^18^F-FDG PET/CT guided the treatment in these patients by evaluating the local recurrence and metastatic disease.

^18^F-FDG PET/CT detected more nodal lesions than CT in 10 staged patients in our study. ^18^F-FDG PET/CT also increased the stage in these patients by detecting multiple lesions in the skeletal system and bone marrow. So, it has been confirmed that ^18^F-FDG PET/CT has addictive effects on the outcomes and the prognosis of patients.

Despite the above-mentioned beneficial roles of ^18^F-FDG PET/CT in malignancy, it has some limitations. Level of radiation dose is a severe problem in children. Lack of simultaneous data acquisition causes image artifacts because of patient movement. Another drawback is that CT provides only limited soft tissue contrast. These problems could be overcome by integrating the PET detectors into MR scanner. Dose reductions of up to 73% have been reported when performing PET/MRI instead of ^18^F-FDG PET/CT because of lack of the CT component, and decreasing the amount of PET tracer administered (because of longer imaging times in PET/MRI) could further reduce the radiation dose (63). Other advantage of PET/MRI is improved soft tissue contrast. Improved soft tissue contrast of MRI leads to improved localization of PET tracer uptake (64). Although ^18^F-FDG PET/CT remains the mainstay for functional imaging of oncologic and neurologic processes in children, early experience shows that PET/MRI has great potential in diagnostic algorithms of several pediatric diseases.

The acquisition parameters for the CT portion of the scan should be tailored to the patient’s size. CT parameters were obtained with ultra-low dose (80 kVp, 5 mAs, and 1.5:1 pitch) in our study. Decreasing the absorbed radiation dose without compromising the image quality can be provided by reducing milliamperes proportionately. This modification results in lower exposed radiation dose in ^18^F-FDG PET/CT than the diagnostic CT. Combination of ^18^F-FDG PET/CT and diagnostic CT has been reported to be used in the literature to prevent doubled radiation exposure to the patient (65). The follow-up of the patients can be performed reliably with ^18^F-FDG PET/CT in order to further reduce the radiation exposure.

## Conclusion

To conclude, ^18^F-FDG PET/CT provides important information for the staging, restaging, response to treatment, and detection of metastatic disease, but it has limited contribution to early diagnosis in childhood tumors particularly in lymphoma, primary bone, and soft tissue tumors. It is a non-invasive imaging method that reflects both the metabolic features and the structural status of the tumors. As the preparation and image interpretation of the pediatric patients differ from adults, these procedures should be performed with specific information and experience on this age group. It should also be noted that indications of ^18^F-FDG PET/CT must be considered appropriately since the exposure to radiation in children has more severe consequences than the adults.

## Figures and Tables

**Table 1:**
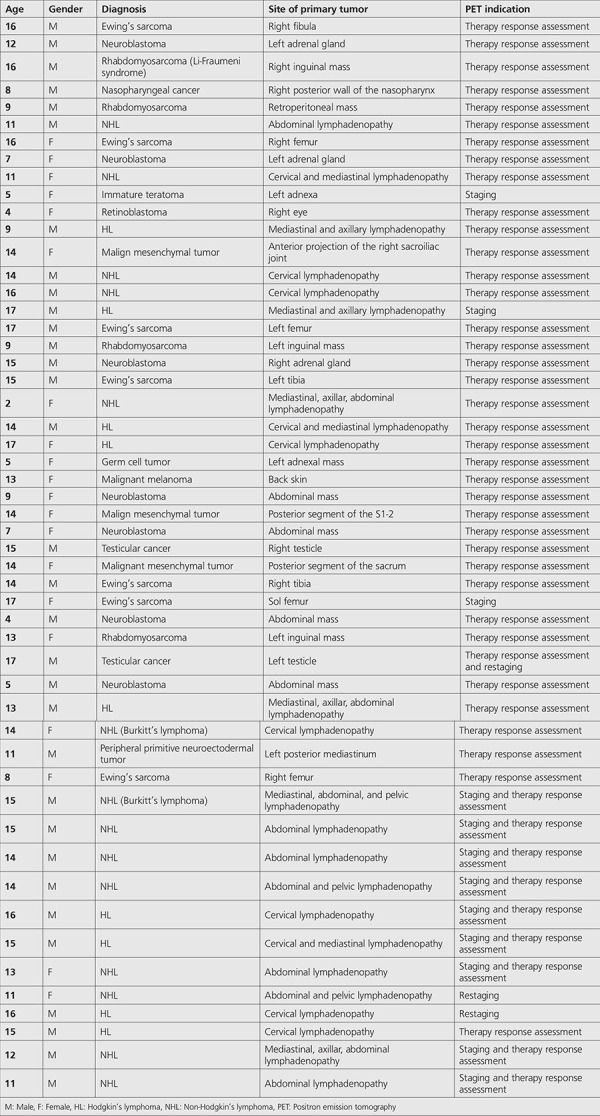
Patient characteristics

**Table 2:**
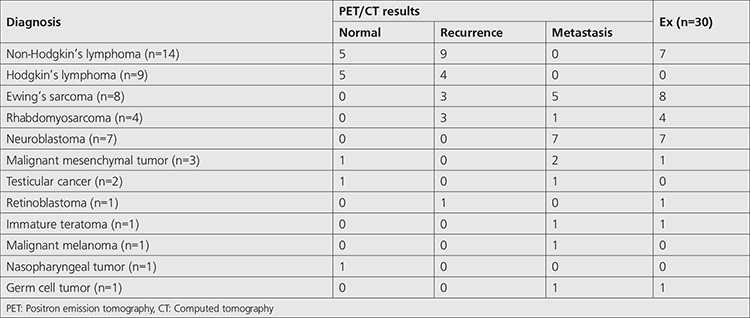
Cancer types, numbers, and follow-up results of all patients
